# Supplementary material for: A Randomized Trial on Resveratrol Supplement Affecting Lipid Profile and Other Metabolic Markers in Subjects with Dyslipidemia
Source: Nutrients. 2023 Jan 17;15(3):492. doi: 10.3390/nu15030492 (PMC9921501; doi:10.3390/nu15030492)
Supplement: Supplementary file 1 [file nutrients-15-00492-s001.zip › nutrients-2155400-supplementary.pdf]

nutrients

Supplementary Materials

A Randomized Trial on Resveratrol Supplement Affecting Lipid Profile and Other Metabolic Markers in Subjects with Dyslipidemia

Table S1. Certificate of analysis for resveratrol.

MegaResveratrol

Certificate of Analysis

**Product Name**	Resveratrol	**Manufacture Date**	March 18, 2021
**Batch No.**	MR210318	**Certificate Date**	March 22, 2021
**Batch Quantity**	100KG	**Expiration Date**	March 17, 2023
**Storage Condition**	Store in cool & dry place, Keep away from strong light and heat		

Item	Specification	Result	Method
**Basic Product Information**			
Genus and Species	*Pohygonum cuspidatum Sieb et Zucc.*	Conform	/
Part of the Plant	Root	Conform	/
**Marker Compounds**			
Assay (Trans-resveratrol)	>99.0%	99.68%	HPLC
Emodin	<0.07%	0.01%	HPLC
**Organoleptic Data**			
Appearance	Fine Powder	Conform	NLS-QCS-1008
Color	Off-white to light yellow	Conform	GB/T 5492-2008
Odor	Characteristic	Conform	GB/T 5492-2008
Taste	Characteristic	Conform	GB/T 5492-2008
**Process Data**			
Method of Processing	Extraction	Conform	/
Solvent(s) Used	Ethanol & Water	Conform	/
Drying Method	Vacuum drying	Conform	/
Excipient	None	Conform	/
Ratio of Extraction	100:1	Conform	/
**Physical Characteristics**			
Melting point	253℃~255℃	254.3℃	GB21781-2008T
Solubility	Soluble in Alcohol	Conform	Visual
Particle Size Analysis	d(10%)<br>d(50%)<br>d(90%)<br>Surface area mean diameter D [3.2]<br>Volume mean diameter D [4.3]<br>Size range	0.681 μm<br>1.420 μm<br>3.492 μm<br>4.176 μm<br>4.023 μm<br>0.018-108.0 μm	GB/T 5507-2008
Loss on drying	<1.0%	0.42%	GB/T 14769-1993
Ash Content	<1.0%	0.33%	AOAC 942.05, 18th
Solvent Residue	Eur Pharm	190ppm	GJ-QCS-1007
**Heavy Metals**			
Total Heavy Metals	<10 ppm	Conform	USP <231>, method II
As	<2.0 ppm	Conform	AOAC 986.15, 18th
Pb	<2.0 ppm	Conform	AOAC 986.15, 18th
Cd	<0.5 ppm	Conform	AOAC 971.21, 18th
**Pesticide Residue**			
666	<0.2ppm	Conform	GB/T5009.19-1996
DDT	<0.2ppm	Conform	GB/T5009.19-1996
**Microbiology**			
Total Plate Count	<1,000cfu/g	80cfu/g	AOAC 990.12, 18th
Total Yeast & Mold	<100cfu/g	20cfu/g	FDA (BAM) Chapter 18, 8th Ed.
E. Coli	Negative	Negative	AOAC 997.11, 18th
Salmonella	Negative	Negative	FDA (BAM) Chapter 5, 8th Ed.

60 Newtown Rd.
MEGA RESVERATROL

Candlewood Stars Inc., Mega Resveratrol, 60 Newtown Rd. Suite 32, Danbury, CT 06810 USA
Tel: 203 794-4030 Toll Free: 877 909-MEGA Fax: 203 798-8287 Email: info@megaresveratrol.com

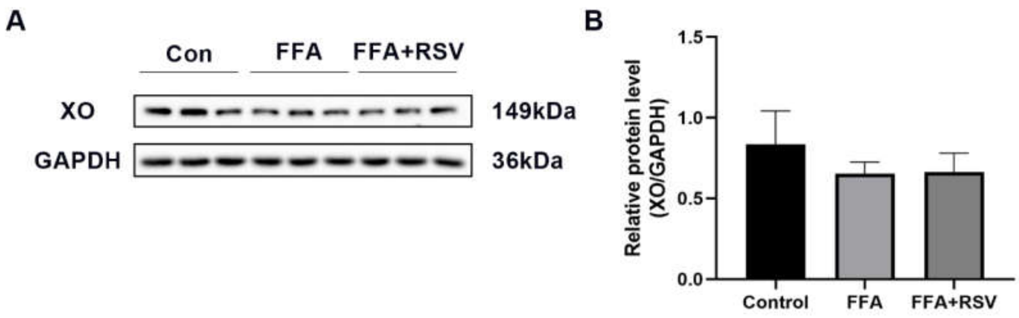

Figure S1. Effect of resveratrol on the protein expression levels of XO in HepG2 cells. Data are expressed as the mean ± SD from the triplicate wells for 3 experiments. (**A**) Western blot analysis of XO expression. (**B**) Relative protein expression levels of XO normalized to GAPDH. FFA, free fatty acids; RSV, resveratrol; UA, uric acid; XO, xanthine oxidase.
